# Resting-State Functional Magnetic Resonance Imaging for Language Preoperative Planning

**DOI:** 10.3389/fnhum.2016.00011

**Published:** 2016-02-01

**Authors:** Paulo Branco, Daniela Seixas, Sabine Deprez, Silvia Kovacs, Ronald Peeters, São L. Castro, Stefan Sunaert

**Affiliations:** ^1^Center for Psychology and Faculty of Psychology and Educational Sciences, University of PortoPorto, Portugal; ^2^Department of Experimental Biology, Faculty of Medicine of Porto UniversityPorto, Portugal; ^3^Department of Imaging, Centro Hospitalar de Vila Nova de Gaia/EspinhoVila Nova de Gaia, Portugal; ^4^Translational MRI, Department of Imaging and Pathology, Katholieke Universiteit Leuven – University of LeuvenLeuven, Belgium; ^5^Department of Radiology, University Hospitals LeuvenLeuven, Belgium; ^6^Medical Imaging Research Center, Katholieke Universiteit Leuven – University Hospitals LeuvenLeuven, Belgium

**Keywords:** fMRI language mapping, resting-state fMRI, independent component analysis, functional MRI, neurosurgery

## Abstract

Functional magnetic resonance imaging (fMRI) is a well-known non-invasive technique for the study of brain function. One of its most common clinical applications is preoperative language mapping, essential for the preservation of function in neurosurgical patients. Typically, fMRI is used to track task-related activity, but poor task performance and movement artifacts can be critical limitations in clinical settings. Recent advances in resting-state protocols open new possibilities for pre-surgical mapping of language potentially overcoming these limitations. To test the feasibility of using resting-state fMRI instead of conventional active task-based protocols, we compared results from fifteen patients with brain lesions while performing a verb-to-noun generation task and while at rest. Task-activity was measured using a general linear model analysis and independent component analysis (ICA). Resting-state networks were extracted using ICA and further classified in two ways: manually by an expert and by using an automated template matching procedure. The results revealed that the automated classification procedure correctly identified language networks as compared to the expert manual classification. We found a good overlay between task-related activity and resting-state language maps, particularly within the language regions of interest. Furthermore, resting-state language maps were as sensitive as task-related maps, and had higher specificity. Our findings suggest that resting-state protocols may be suitable to map language networks in a quick and clinically efficient way.

## Introduction

Functional magnetic resonance imaging (fMRI) is a well-known imaging tool that is used to identify brain regions up to the millimeter scale that exert an increased blood oxygen level dependent (BOLD) response when subjects execute a task compared to control tasks (Ogawa et al., 1990; Binder, 2006). Due to the high spatial resolution and non-invasiveness of the technique, the popularity of fMRI for the study of brain function has greatly increased during the last decade particularly in language research (Price, 2012). One of the most common clinical applications of fMRI is pre-surgical mapping of language (e.g., Binder et al., 1996; Roux et al., 2003; Stippich et al., 2007). By having the subject perform simple tasks in the scanner, fMRI has the potential to localize brain regions involved in language processing, allowing neurosurgeons to spare eloquent brain tissue in invasive procedures such as tumor removal or surgery for refractory temporal lobe epilepsy. Although widely used, language mapping in the brain for pre-surgical planning faces several methodological challenges: variability of results with different language tasks (Binder et al., 2008), susceptibility to poor task performance (Lee et al., 1999; Price et al., 2006), motion artifacts and low signal-to-noise ratio (Seixas and Lima, 2011), among others (for a review see Sunaert, 2006). As a consequence, the specificity and sensitivity of task-based fMRI when compared to the clinical gold-standard – direct cortical stimulation (DCS) – is presently rather modest (for a review, see Giussani et al., 2010).

More recently so-called resting-state fMRI has been discovered. Functional MR images acquired while subjects are at rest (not performing any task) show low frequency (<0.1 Hz) BOLD signal changes in several spatially distinct brain networks (Damoiseaux et al., 2006; Fox and Raichle, 2007; Fox and Greicius, 2010). By using correlation or blind-source separation of these signals, well-known functional networks can be extracted from resting-state fMRI data, such as the auditory, the visual and the sensory-motor networks (Beckmann et al., 2005; Smith et al., 2009). The rationale is that brain regions that are intrinsically and functionally connected share similar time-courses and can, therefore, be separated from others and proven statistically independent (Beckmann and Smith, 2004; Deco et al., 2011). Preliminary results show that resting-state fMRI can also identify the language network (Mitchell et al., 2013; Tie et al., 2014; Zhu et al., 2014).

Using resting-state fMRI protocols instead of task-fMRI has several advantages. In task-fMRI the percentage of BOLD signal increase between two conditions is generally small; in resting-state protocols, however, the BOLD signal oscillations proper are studied and these provide up to three times higher signal-to-noise ratio than task-related signal increases (Fox and Greicius, 2010). Another key advantage of resting-state techniques is the absence of a task. While healthy subjects usually perform well and collaborate efficiently in the required tasks, this can be problematic for subjects with brain lesions (Lee et al., 1999; Price et al., 2006). The majority of patients eligible for pre-surgical planning have neurological deficits that hinder task performance, with obvious consequences for the fMRI results obtained with this approach. The absence of a task also reduces activity confounds such as complementary task-related activity (e.g., visual activity in a language task) that can influence the observed brain responses with fMRI (Fox and Greicius, 2010). Finally, a resting-state examination is more time-efficient than a task-based one, because the imaging protocol is typically faster and the collected data serves multiple mapping purposes (Lang et al., 2014), thus fitting better into the usually limited patient scanning schedule.

The feasibility of using single-subject resting-state fMRI for mapping brain functions has recently received more attention in the scientific literature. In motor mapping, a few studies have shown that task-based statistical maps and resting-state maps are quite similar (e.g., Kokkonen et al., 2009; Shimony et al., 2009; Zhang et al., 2009; Rosazza et al., 2014). In language mapping, however, not much is known. A recent study with healthy subjects (Tie et al., 2014) showed that blind-source separation of signals by independent component analysis (ICA) followed by an automated classification of language components resulted in a good overlap between resting-state language networks and fMRI task-based activity. Although these findings indicate that resting-state protocols may be suitable for language mapping in healthy volunteers, it is still unknown if this process can be used in surgical patients as well. The classification and extraction of independent components (ICs) in patients presents several challenges: the distorted anatomy may hinder the classification of ICs; patients tend to produce more movement artifacts (Lee et al., 1999); and functional connectivity could be affected by the pathology itself (e.g., Waites et al., 2006). An encouraging first study with epileptic and tumor patients showed a fair sensitivity and specificity of resting-state fMRI language mapping as compared to DCS by using machine learning to extract the language networks (Mitchell et al., 2013). The machine learning technique was able to identify the language networks, but it required a training phase and *a priori* knowledge about them. This is technically demanding and may prove to be difficult to implement, particularly when dealing with plasticity effects or the developing brain. Using ICA, however, may overcome some limitations of the machine learning technique by extracting language networks without an *a priori* hypothesis. This would reduce the potential bias associated with training in machine learning.

Finally, although resting-state fMRI seems a promising method, it remains to be tested whether the observed networks are specific and accurate enough for language mapping in a clinical setting. Furthermore, task-related activity might be better suited to localize specific areas involved in subdomains of language, such as syntax or semantics (Fedorenko et al., 2010; Hope et al., 2014). Only by comparing resting-state networks with task-related activity can we ascertain whether networks extracted through functional connectivity tap into the complex activations subserving task execution, and are effective for mapping language in the brain.

The purpose of this study is to investigate if resting-state fMRI can replace conventional task-based approaches for language mapping in pre-surgical patients. We used a data-driven method that requires no *a priori* hypothesis (ICA) to extract resting-state language networks, and examined the overlay between task-based and resting-state language maps in a cohort of patients with brain lesions referred by neurosurgeons for preoperative mapping.

## Materials and Methods

### Participants

Fifteen patients (mean age 37.5 ± 12.4 years, 12 men) participated in the study. Subjects with brain tumors and epilepsy were included in the group. Lesion information and demographic data are described in **Table 1**. Seven subjects were left-handed; however, all subjects were left lateralized for language as determined by the local standard procedure in which a language laterality index is computed from task fMRI data and a classification is made derived from this index supplemented by visual inspection. The study was approved by the local ethics committee, with informed consent. The data was obtained as part of the routine clinical pre-surgical workup of the patients, which included structural imaging and fMRI.

**Table 1:** Patient demographic and clinical data.

Case	Age	Gender	Handedness	Brain laterality^∗^	Pathology	Lesion type	Lesion side	Lesion location
1	27	M	L	L + 0.71	Epilepsy	MRI undetectable lesion	R	Frontal EEG abnormalities
2	46	M	R	L + 0.29	Tumor	GBM	L	Fronto-rolandic region
3	44	M	L	L + 0.18	Tumor	Glioma grade 3	L	Superior temporal gyrus
4	19	M	L	L + 0.29	Epilepsy	MRI undetectable lesion	R	Right temporo-parietal EEG abnormalities
5	42	M	R	L + 0.34	Tumor	GBM	L	Left precentral gyrus
6	47	M	L	L + 0.54	Tumor	Recurrent GBM	R	Temporo-parietal junction
7	34	M	R	L + 0.56	Tumor	Cavernoma	R	Hippocampus and parahippocampal gyrus
8	36	M	L	L + 0.19	Tumor	LGG	L	Temporo-occipital junction
9	65	F	R	L + 0.22	Tumor	Choroid plexus papilloma	L	Atrium of the lateral ventricle
10	43	M	R	L + 0.50	Tumor	Oligodendroglioma grade 2	L	Superior temporal gyrus
11	33	M	R	L + 0.72	Tumor	Oligodendroglioma grade 2	L	Anterior temporal lobe
12	39	F	L	L + 0.51	Tumor	Ganglioglioma grade 1 and cortical dysplasia	L	Mesial temporal lobe
13	44	M	L	L + 0.49	Tumor	Recurrent GBM	R	Temporal lobe
14	30	F	R	L + 0.22	Tumor	Oligodendroglioma grade 2	R	Temporo-opercular region
15	14	M	R	L + 0.39	Epilepsy	Focal stroke with reactive gliosis	L	Inferior frontal gyrus

*^∗^Brain laterality was determined independently by the medical staff, via analysis of a laterality index for each subject supplemented with visual inspection. The laterality index (-1 to 1, negative values for right- and positive values for left-lateralization) was quantified by calculating the proportion of left hemisphere to right-hemisphere significant voxels in the task-based protocol with a threshold of z > 4.*

### Magnetic Resonance Imaging

Subjects were scanned in a Philips (Best, The Netherlands) 3 Tesla Achieva scanner with a 32-channel array head coil. All subjects performed resting-state and task-fMRI protocols in the same session as part of their routine examination. For the task-based fMRI session a T2^∗^ weighted gradient echo-echo planar imaging (GE-EPI) sequence was used with the following parameters: repetition time (TR) = 3000 ms; echo time (TE) = 33 ms; matrix size = 80 × 80; field-of-view (FOV) = 230 mm; flip angle 90°; slice thickness 4 mm, no gap; in-plane pixel size = 2.88 mm × 2.88 mm; and axial slices = 35. A total of 160 functional volumes per subject were collected, divided in two runs of 80 volumes each. The accumulated duration of both runs was 8 min. For resting-state fMRI, data was acquired using a T2^∗^ weighted GE-EPI sequence with the following parameters: TR = 1700 ms; TE = 33 ms; matrix size = 64 × 64, FOV = 230 mm; flip angle 90°; slice thickness = 4 mm, no gap; in-plane pixel size = 3.59 mm × 3.59 mm; and axial slices = 32. Two hundred and fifty functional volumes were obtained in 7 min 10 s.

A high-resolution T1-weighted image was also acquired for registration purposes, using a coronal three-dimensional turbo field echo sequence with the following parameters: 182 contiguous coronal slices covering the whole brain and brainstem, slice thickness = 1.2 mm; TR = 9.7 ms; TE = 4.6 ms; matrix size = 256 × 256; FOV = 250 mm × 250 mm; in-plane pixel size = 0.98 mm × 0.98 mm; acquisition time, 6 min 38 s.

### Procedure

For the resting-state fMRI protocol, all subjects were instructed to relax (but not sleep) in the scanner with their eyes closed, while thinking of nothing in particular. For task-based fMRI, a well-established (Petersen et al., 1988; Benson et al., 1999) verb-to-noun generation paradigm was used. Subjects were presented with a visual noun and were asked to covertly generate one or more related verbs (e.g., ‘glass’ – ‘drink’).

Stimuli were presented in a block design, alternating eight task epochs (30 s each, 3 s per noun) with eight periods of rest (30 s each). During rest epochs, subjects viewed a series of unpronounceable visual symbols in the center of the screen (e.g., #-°/*). Visual stimuli were presented on a MRI-compatible screen using Presentation software (version 14.1, Neurobehavioral Systems Inc., Albany, CA, USA). Subjects’ ability to perform the task was assessed before scanning. Due to task performance issues (slow responses), one subject did the task at a TR of 5 s instead of 3 (slightly extended response time).

### Data Analysis

**FIGURE 1:**
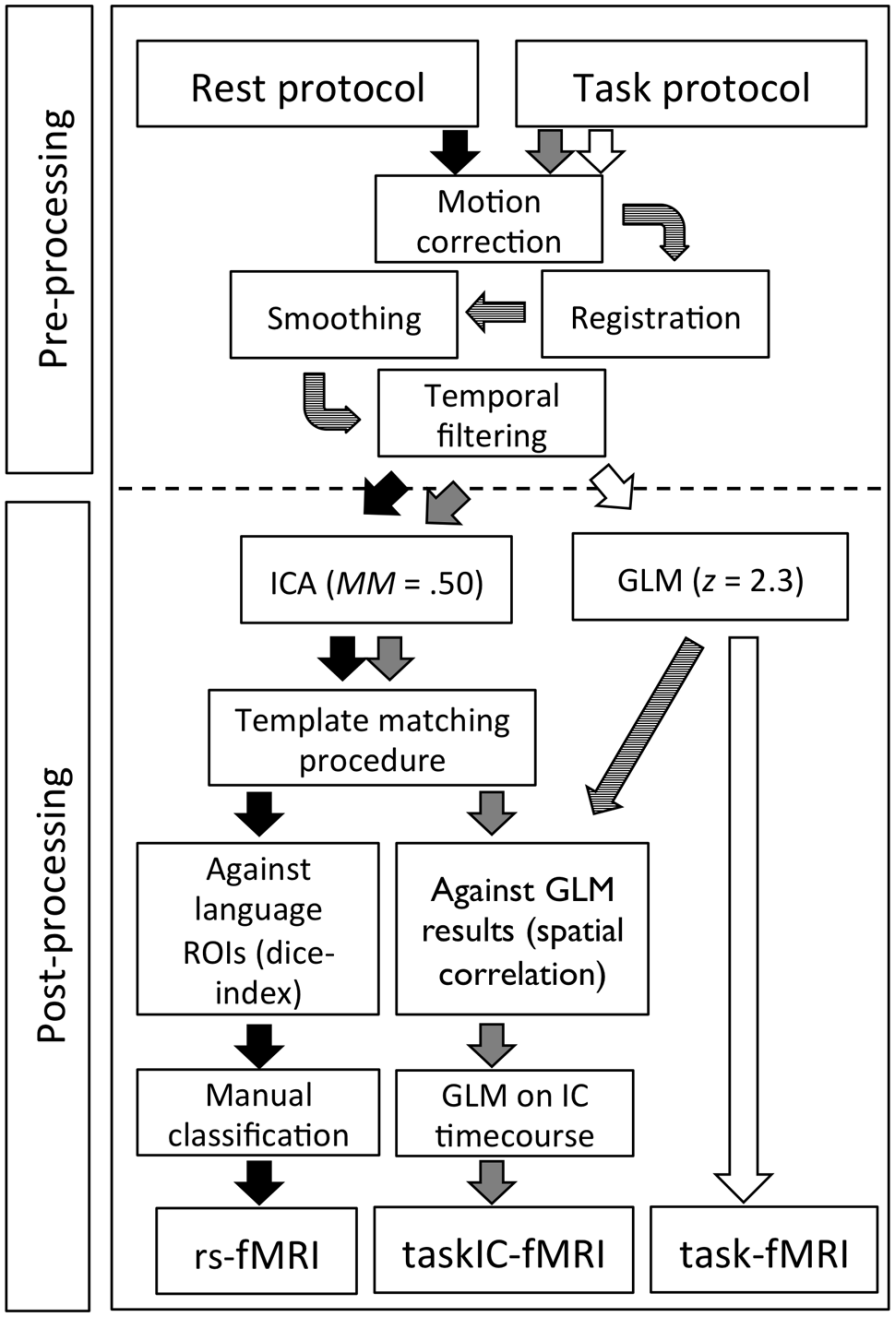
**Overview of the image pre-processing and post-processing steps for resting-state rs-fMRI, taskIC-fMRI and task-fMRI**.

All analyses were done using the Oxford Centre for Functional Magnetic Resonance Imaging of the Brain Software Library (FMRIB, Oxford UK; FSL version 5.0.4). An overview of the pre and post-processing steps for resting-state and task-based protocols can be found in **Figure 1**. Similar pipelines were used for pre-processing resting-state and task-based fMRI data: motion correction was performed using MCFLIRT (Jenkinson et al., 2002); functional data was transformed into subjects’ structural space and resampled to 2 mm × 2 mm × 2 mm voxel size using FLIRT (Jenkinson et al., 2002). Due to brain deformations caused by lesions, registrations between the structural and functional images were performed using rigid body transformation with 6 degrees-of-freedom (DOF) and later manually improved if necessary. Non-brain tissue was removed with BET (Smith, 2002); spatial smoothing was applied using a 6 mm Full Width at Half Maximum (FWHM) Gaussian kernel; and high-pass temporal filtering was performed at 60 s FWHM for task-based fMRI and 100 s FWHM for resting-state fMRI. We also obtained transformation matrices from the high-resolution T1 images to standard Montreal Neurological Institute (MNI) space using a 12 DOF affine linear registration in order to convert regions-of-interest (ROI) to structural space and to generate group images. All analyses were performed independently for each subject.

To extract language networks from resting-state fMRI data (rs-fMRI hereafter), we used Probabilistic Independent Component Analysis (PICA) through single-session Multivariate Exploratory Linear Optimized Decomposition into Independent Components (MELODIC version 3.13, Beckmann and Smith, 2004). Voxels outside the brain were removed and the signal was demeaned and normalized for each voxel. Data sets were then whitened and projected to an n-dimensional subspace using PICA. The number of components (mean ICs = 66.3 ± 9.9, range 53–82) was estimated using FSL default Laplacian approximation (Minka, 2000; Beckmann and Smith, 2004). The IC maps were thresholded using a mixture-model and alternative hypothesis testing approach, with the threshold parameter set to 0.5 for an equal weight in false positives and false negatives (Woolrich et al., 2005).

For post-processing of task-based fMRI (task-fMRI hereafter), FEAT (FMRI Expert Analysis Tool, Smith et al., 2004) version 6 was used. FILM pre-whitening was used on the EPI images and a simple contrast between block conditions convolved with the build-in hrf was performed for each run, including the motion parameters generated in the preprocessing steps as additional regression parameters for the general linear model (GLM). The two acquired runs for each subject were then passed on to a higher-level fixed-effects GLM using a *z* > 2.3 threshold and a Gaussian Field Theory corrected cluster *p* threshold of 0.05. In order to examine the task results using multivariate analysis (analogous to the analysis of resting-state data), we further performed PICA on the task fMRI data (taskIC-fMRI hereafter) using MELODIC and the same procedure and threshold as for the resting-state data. This allowed to identify non-model driven activation maps that reduced artefactual activation as well as non-language related activity in the task-based fMRI, potentially increasing sensitivity and specificity (Tie et al., 2008). For each subject, task-based ICs were identified by doing a spatial cross-correlation between all resulting component maps and the GLM results. The highest correlated IC (i.e., the component with the highest spatial resemblance to the GLM results) was selected. To confirm that the selected IC was related to the language task, a GLM analysis was computed on the average BOLD time-course of the component using the same block design model as that for the task-based fMRI. All the identified component time-courses corresponded to the task-model (*p*s < 0.001).

### Classification of Resting-State Components

To identify each subject’s language resting-state component(s), we performed a semi-automated template-matching procedure. Firstly, the total number of components was reduced by excluding components with more than 50% of frequency power above 0.1 Hertz; related components are typically below this range, whereas various scanner or physiological artifacts have high frequencies (De Martino et al., 2007; Mannfolk et al., 2011). This reduced the chance of incorrectly classifying noise components with coincidental spatial coherence with the language areas. Secondly, we calculated the overlay of each remaining component against the predefined language ROIs provided by Fedorenko et al. (2010). These ROIs were validated in a set of healthy subjects and represent the major language areas derived from single-subject analysis (Fedorenko et al., 2010). They include thirteen brain areas: in the left hemisphere, the angular gyrus (AG), the superior frontal gyrus (SFG), the medial frontal gyrus (MFG), the inferior frontal gyrus (IFG), the inferior frontal gyrus pars orbitalis (IFGorb), the left cerebellum, the middle posterior temporal (MidPostTemp), posterior temporal (PostTemp), middle anterior temporal (MidAntTemp) and anterior temporal (AntTemp) regions; and in the right hemisphere, the middle anterior temporal (rightMidAntTemp) and middle posterior temporal (rightMidPostTemp) regions, as well as the right cerebellum. Because of the number and large extent of these ROIs, this classification method has the advantage of being specific enough to capture language-related areas at the single subject level, and yet sufficiently comprehensive to compensate for the potential dislocation of language areas secondary to brain lesions. The ROIs were combined into a single mask and transformed from MNI space into each subject’s structural space through inverse linear transformations obtained from the initial transformation matrices.

To measure the similarity between IC masks and the language ROIs, we computed the Dice coefficient using the same equation as Tie et al. (2014)^1^. The Dice coefficient varies between 0 and 1, and provides an objective measure of overlap between two masks, X and Y. Higher values represent more similarity. We calculated Dice coefficients between each IC and the language ROIs, and ranked the IC components according to the value of the Dice coefficient from highest to lowest. Due to the high number of components extracted, only the top 10 ranked components for each of the 15 subjects were used in subsequent analyses (150 total, 10 components × 15 subjects). These IC components were examined by an expert neuroradiologist (D.S.) with extensive practice in preoperative mapping of language with fMRI, and blinded to the ranking obtained through the template-matching procedure and to the task-based results. The neuroradiologist classified how confident she was that each component was mapping language using a 1–5 Likert scale (1 = it is very unlikely that this component is related to language; 5 = it is very likely that this component is related to language). There was no preset limit to the confidence ratings in the sense that for a single subject more than one or two components could be given maximum confidence.

Classification accuracy was calculated by comparing the ICs identified in the template-matching procedure and the component(s) with the highest confidence rating in the expert manual classification. The expert-selected component was used as ground-truth and the ICs were classified with regard to the position on the ranking provided by the classification procedure (First ranked component, second ranked component, third ranked component, fourth or worse). A perfect accuracy would mean that all first ranked components in the template-matching procedure corresponded to the highest-rated IC by the expert.

### Comparison Between Task-Related Activity and Resting-State Networks

To assess the overlay of task-related areas with resting-state areas, we calculated the Dice coefficient using the masks from the task-fMRI analyses and the resting-state components. In order to have comparable analysis methods and statistical thresholding, we added a comparison between resting-state maps and a multivariate analysis of task-activity, taskIC-fMRI (see **Figure 1**). Dice coefficients were obtained for the whole-brain and within the language ROIs. Sensitivity and specificity were also examined for resting-state and task-fMRI methods. To do so, we used the predefined language ROIs as ground-truth for the critical language regions. As in Tie et al. (2014), specificity was measured as the percentage of voxels in each mask falling within the language ROIs, and sensitivity as the total number of voxels of each mask within the language ROIs.

To investigate the consistency of resting-state results and task results across subjects, we created a probability overlap map. This was achieved by transforming the binary masks of rs-fMRI, task-fMRI and taskIC-fMRI to MNI standard space and adding each subject into a single volume. The resulting image shows the number of subjects with significant activity for each voxel, from 1 to 15. This allowed us to examine the relevant brain regions that were identified across subjects in each technique, and to compare them between resting-state and task-based methods. Finally, due to the variability in the statistical thresholds of single subjects, we examined the effect of different thresholds in the Dice coefficient by calculating Dice coefficients with increasing thresholds in steps of 0.25, from *z* = 2 to *z* = 6, for resting-state and for task data. We also calculated sensitivity and specificity values at different thresholds from *z* = 2 to *z* = 6 in steps of 1.

### Effects of ICA Dimensionality on Language Resting-State Networks

One of the challenges of the ICA procedure is the selection of the optimal dimensionality such that the risk of splitting a higher-order network into several sub-networks is avoided (Smith et al., 2009). To examine the stability of the language IC maps at different dimensionalities, ICA analyses were repeated with a fixed number of ICs, from 20 to 120 ICs, in steps of 10. The corresponding language ICs at each dimensionality were determined by performing a spatial correlation against the original language IC and confirmed by visual inspection. To quantify changes in ICs, the spatial correlation between the original IC and the corresponding IC for each dimensionality was computed, as well as the temporal correlation between the time-courses of both ICs. To obtain a single index of temporal and spatial change, we divided 1 by the product of the spatial and the temporal correlations. This measure captured whether language networks were branched into sub-networks as a consequence of the selected dimensionality. If more than one, networks should merge when enforcing lower dimensionalities (e.g., Smith et al., 2009; Esposito and Goebel, 2011) and this would show up as a marked reduction of spatial and temporal correlations. If the original number of ICs was optimal, no significant changes in spatial and temporal correlations should be observed. Not all detected changes would reflect a merge of language subcomponents, as the language network could also merge with unrelated ICs at lower dimensionalities. Thus, for each dimensionality we examined the ratio between voxels inside and outside language ROIs (specificity) and compared it with the corresponding ratios in the original dimensionality.

## Results

### Expert Classification of Resting-State Language Components

In order to determine whether the expert selected one or more ICs as being confidently related to language, we analyzed the expert’s top-rated component for each subject (e.g., the IC with the highest confidence) and the immediate next best option (e.g., the IC with the second highest confidence). The average confidence of the expert in the IC rated with the highest confidence for all subjects was 4.27 ± 0.8 (mean ± SD, maximum = 5), while the average second best confidence was only 2.53 ± 0.8. Thus, for each subject one IC stood out clearly as the best component in the expert evaluation. Due to the low confidence values for the second-rated IC (and the remaining alternatives), only the component with the highest confidence for each subject was taken as the expert-selected correct resting-state language component. When comparing the expert-selected language component with the first-ranked component from template-matching classification, we obtained an accuracy of 80% — a correct match of 12 out of 15 subjects. The second-ranked template-matching IC agreed with the expert-selected IC for the remaining 20% — three subjects.^2^ Given the good agreement between the expert classification and the IC components, we used the expert-selected ICs in the following analyses.

### Task and Resting-State Language Mapping Results

**FIGURE 2:**
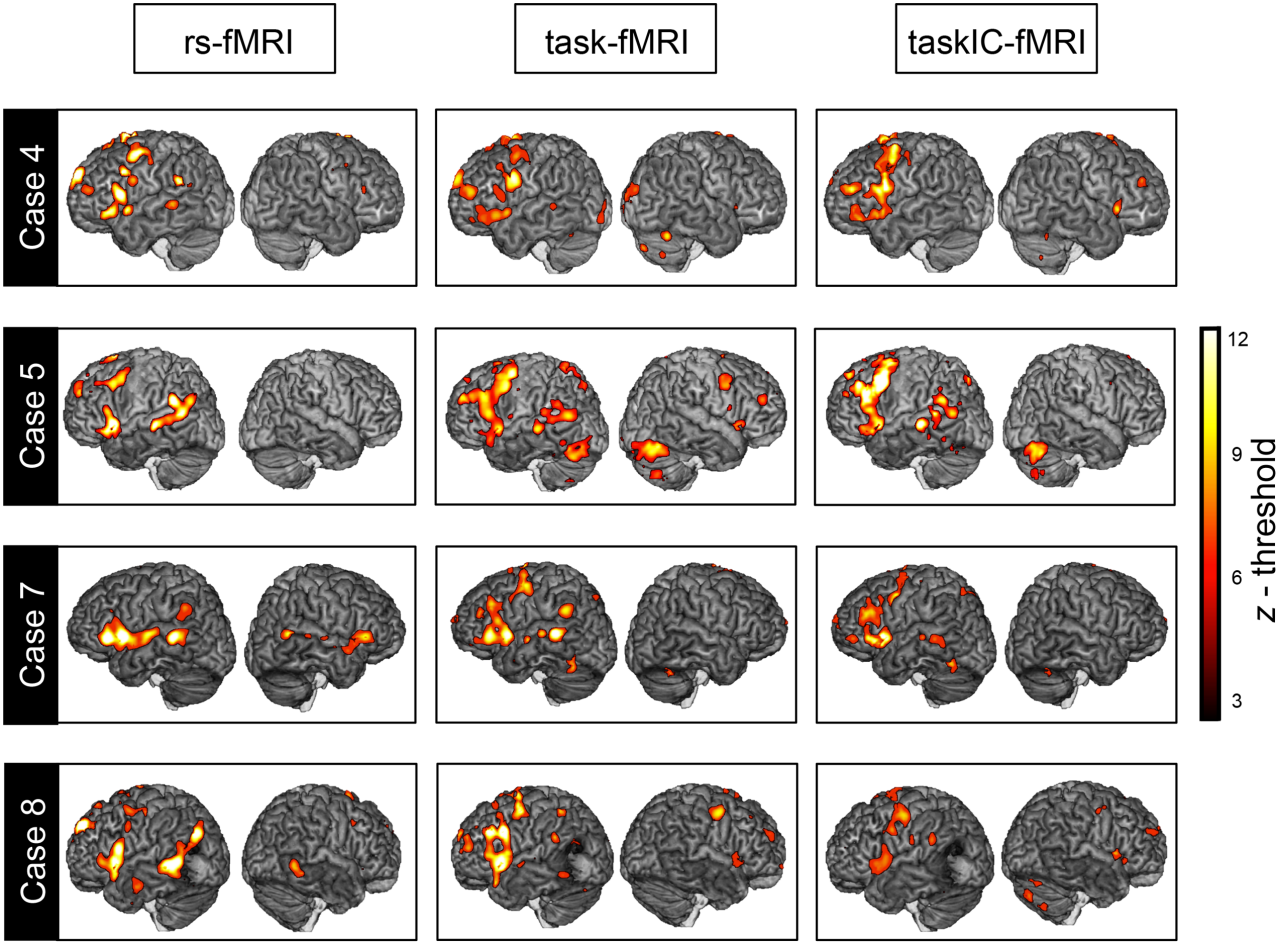
**Overlay between resting-state fMRI, task-fMRI and taskIC-fMRI**. Language maps from representative subjects (Cases 4, 5, 7, and 8, see **Table 1**) with a Dice of 0.356, 0.297, 0.310, and 0.199 respectively, for task-fMRI and 0.340, 0.389, 0.315, and 0.298 respectively, for taskIC-fMRI. Threshold was set at *z* = 3 for illustration purposes.

**Figure 2** illustrates the language mappings obtained via the three methods, rs-fMRI, task-fMRI, and taskIC-fMRI, for four representative subjects (maps from all subjects can be seen in Supplementary Figure S1). The resting-state language map had an average of 16418 ± 3796 voxels. With the task-based GLM method, the average size of the language activations with a threshold of *z* = 2.3 was 37375 voxels ± 18251 and when using ICA with a mixture-modeling threshold of 0.5, the average size of the mask was 29532 ± 9304 voxels. Repeated measures analysis of variance (rmANOVA) on the voxel counts revealed a main effect of method [*F*(1.3,18.2) = 12.72, *p* = 0.001, $\eta_{p}^{2}$ = 0.48]. Resting-state masks had significantly less active voxels than task-based masks, as confirmed by Tukey *post hoc* tests (resting-state vs. task-fMRI, *p* < 0.001, vs. taskIC-fMRI, *p* = 0.011).

### Comparison Between Task and Resting State Language Maps

**Table 2** presents the Dice coefficients for each subject according to method of analysis, separately for the whole brain and for the language ROIs (Fedorenko et al., 2010). In the whole brain analysis, the overlay between resting-state and task-fMRI was on average 0.248 ± 0.075 (mean Dice coefficient). The overlay was significantly higher between resting-state and taskIC-fMRI [Dice = 0.298 ± 0.086, *t*(14) = 2.60, *p* = 0.021, *d* = 0.68].

**Table 2:** Dice coefficients between resting-state and task-based Maps (task-fMRI and taskIC-fMRI) for the whole-brain and within language regions-of-interest (ROI), for each subject.

	rs-fMRI vs. task-fMRI		rs-fMRI vs. taskIC-fMRI	
Subject	Whole brain	ROI	Whole brain	ROI
1	0.218	0.347	0.367	0.619
2	0.227	0.529	0.298	0.523
3	0.191	0.329	0.344	0.500
4	0.356	0.663	0.340	0.652
5	0.297	0.587	0.389	0.587
6	0.220	0.448	0.289	0.490
7	0.310	0.580	0.315	0.486
8	0.199	0.370	0.298	0.498
9	0.274	0.507	0.240	0.308
10	0.400	0.653	0.419	0.641
11	0.129	0.234	0.177	0.260
12	0.247	0.408	0.400	0.567
13	0.197	0.311	0.258	0.469
14	0.149	0.377	0.113	0.251
15	0.304	0.531	0.217	0.360

Mean	0.248	0.458	0.298	0.481
*SD*	0.075	0.131	0.086	0.131

In the language ROIs, the mean Dice coefficient was 0.458 ± 0.131 with task-fMRI, and 0.481 ± 0.131 with taskIC-fMRI. In both cases, it is significantly higher than in the whole-brain analysis [*t*(14) = 11.77, *p* < 0.001, *d* = 3.04 for task-fMRI; *t*(14) = 11.39, *p* < 0.001, *d* = 3.02 for taskIC-fMRI]. Within language ROIs, Dice coefficients were similar in the two task-based methods (*t* < 1).

**FIGURE 3:**
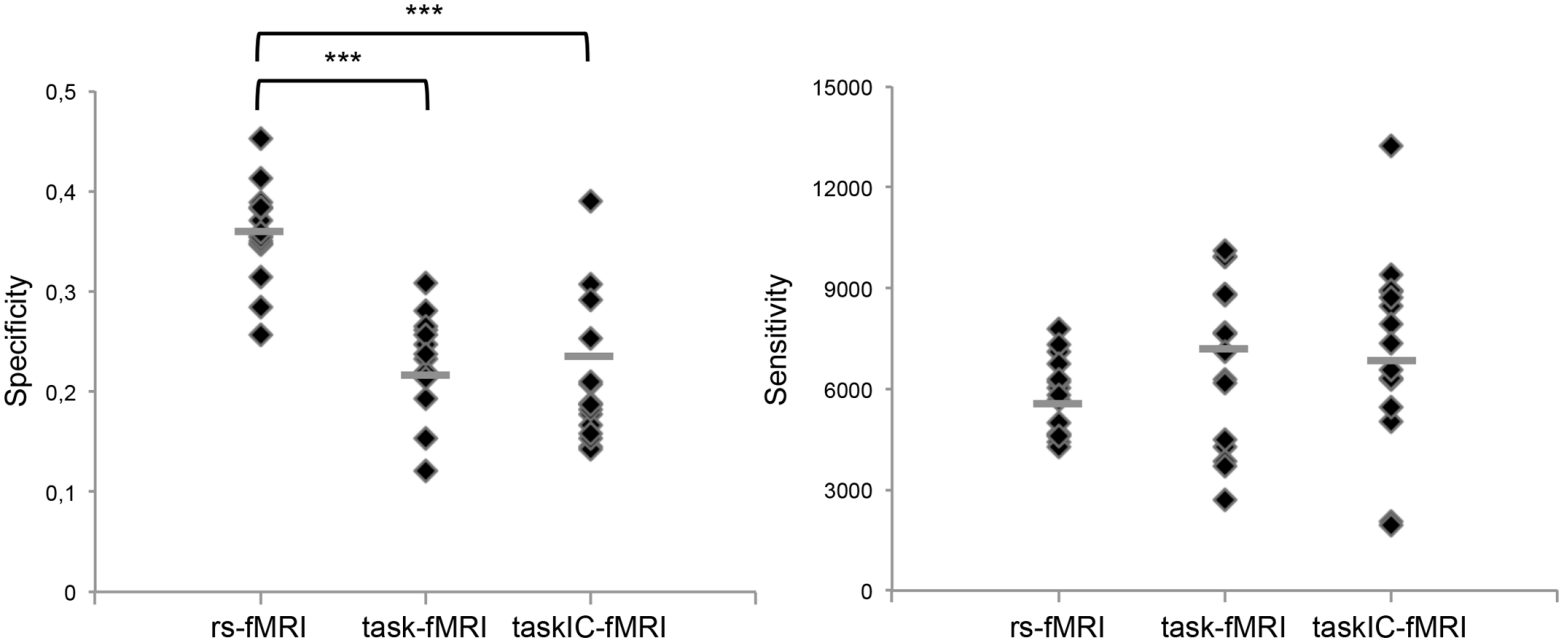
**Specificity in percentage of voxels within language ROIs, and sensitivity in number of voxels within language ROIs, for resting-state fMRI, task-fMRI, taskIC-fMRI, for each subject.** The horizontal line shows the mean value in each case. ^∗∗∗^ = differences at *p* < 0.001.

We found that resting-state networks had higher specificity (percentage of voxels within language ROIs) when compared to task-based fMRI, in both methods for the analysis of the task-based protocols. The percentage of rs-fMRI activated voxels within the functional ROIs was 36 ± 5%. Corresponding values for task-fMRI and for taskIC-fMRI were 21% ± 7 and 23% ± 5, respectively. The effect of method was significant [rmANOVA, *F*(1.7,24.2) = 31.35, *p* < 0.001, $\eta_{p}^{2}$ = 0.69]. *Post hoc* Tukey tests showed that the percentages of activated voxels in both task fMRI methods did not differ (*p* = 0.66), but both were significantly lower than the percentage found in resting-state networks (*p*s < 0.001). Regarding sensitivity (number of activated voxels within the ROIs), the inverse pattern was observed: it was largest in task-fMRI (7106 ± 2866), followed by taskIC-fMRI (6763 ± 2508) and rs-fMRI (5770 ± 1142). However, these differences were not statistically significant [*F*(1.7,24.2) = 1.75, *p* = 0.197]. For an overview of the sensitivity and specificity values for each technique/subject, see **Figure 3**.

**FIGURE 4:**
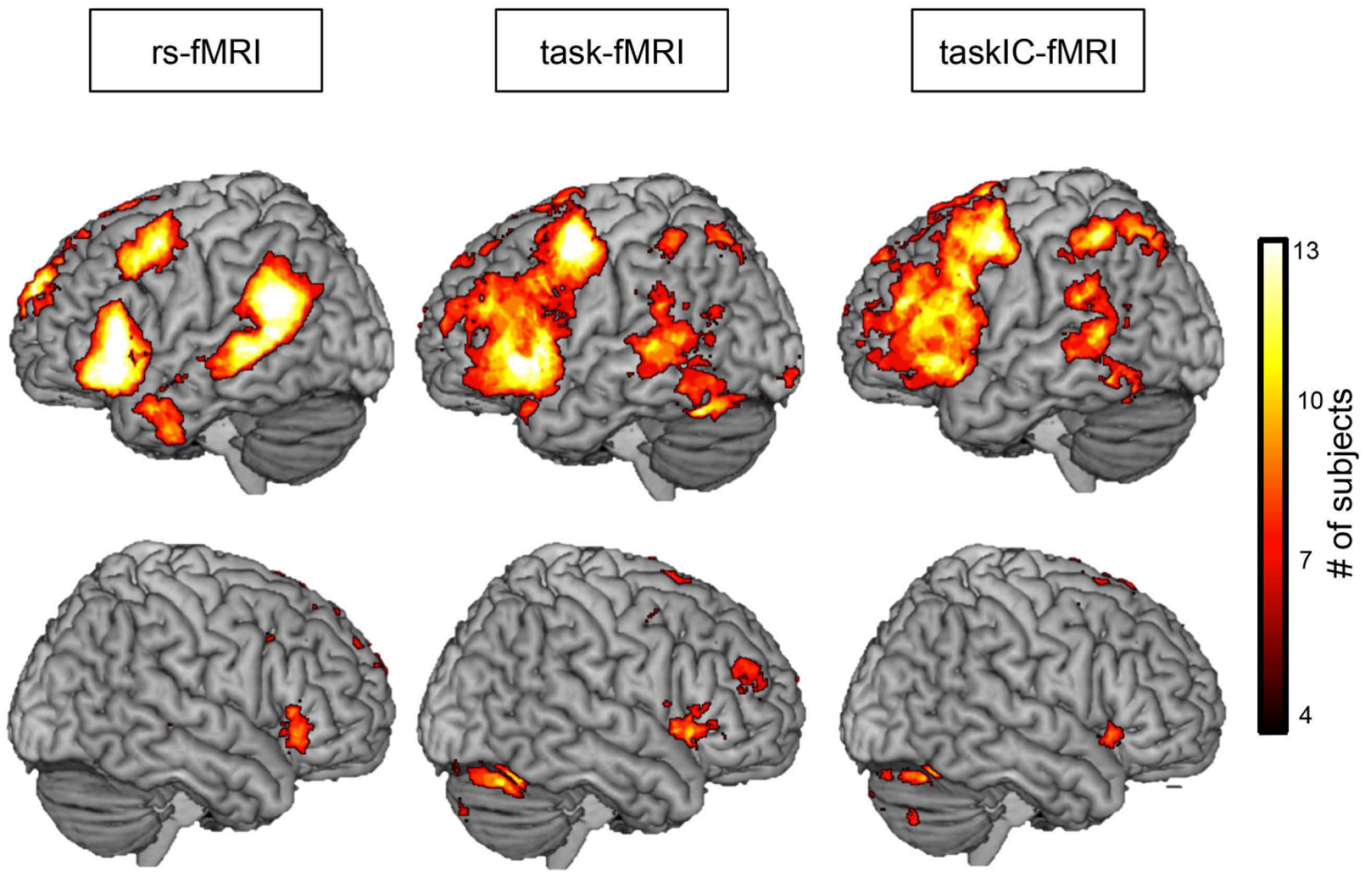
**Probabilistic overlap map across subjects for rs-fMRI, task-fMRI and taskIC-fMRI.** Colors represent the number of subjects with significant activations at each voxel. Images were thresholded between 4 and 13 subjects for better visualization.

**Figure 4** shows the overlay across subjects for the three masks transformed into standard space. Resting-state fMRI exhibited larger coherence across subjects and less spatial extent. Furthermore, activity was more restricted to the critical language regions in rs-fMRI than in both task-fMRI methods, that had larger spatial extent as well as larger variability across subjects.

### Influence of Threshold on Dice, Sensitivity and Specificity

**FIGURE 5:**
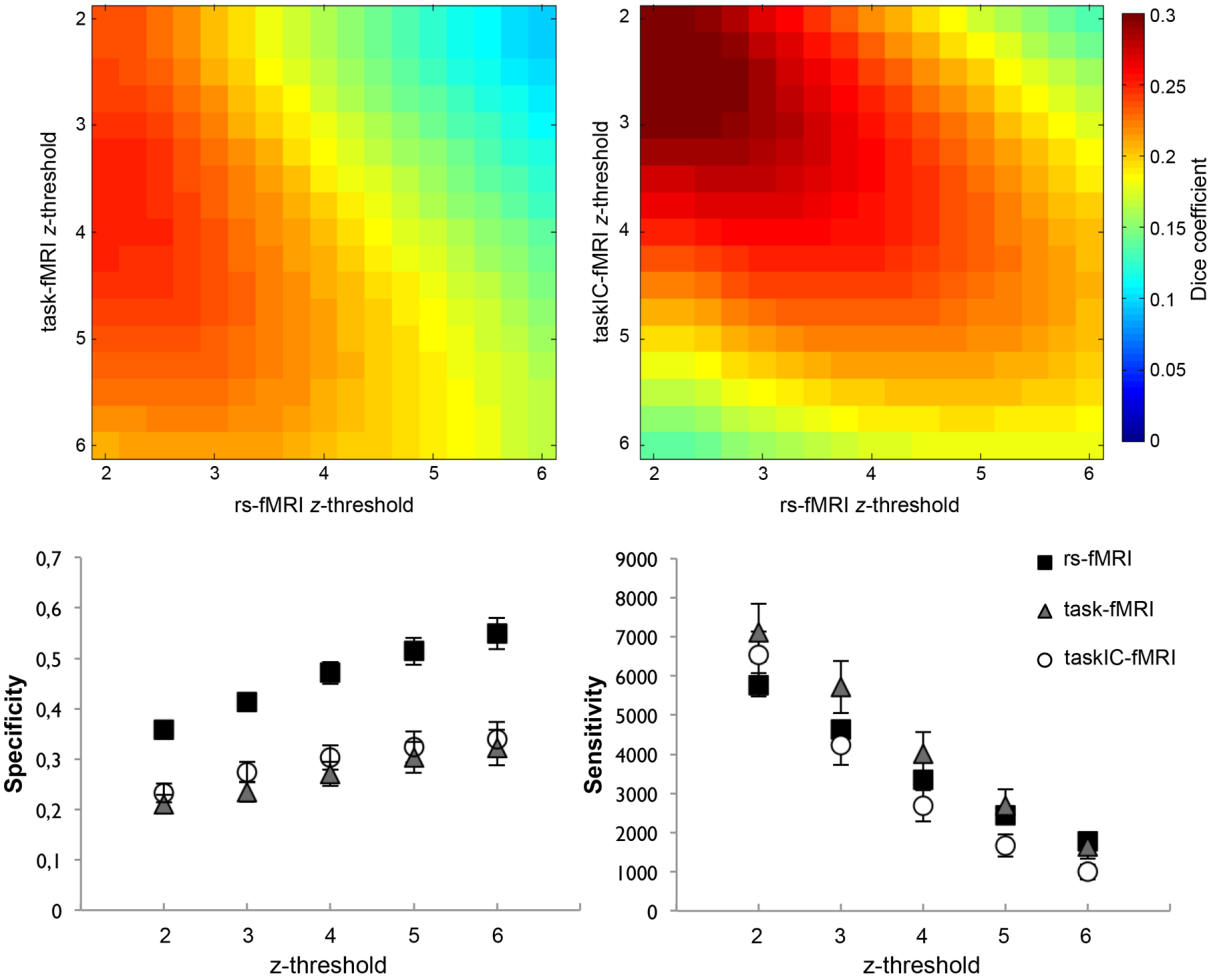
**(Upper panel)** Dice coefficient averages at different thresholds (*z* = 2 to *z* = 6) between rs-fMRI and task-fMRI **(left)**, and between rs-fMRI and taskIC-fMRI **(right)**. Colors represent the average Dice coefficient for all subjects at each threshold. **(Lower panel)** Specificity (left) and sensitivity (right) for rs-fMRI, taskIC-fMRI and task-fMRI at different thresholds (from *z* = 2 to *z* = 6). Vertical bars denote standard errors of the mean.

To assess the influence of the selected threshold on the overlay between task and resting-state, we calculated the Dice values by iteratively increasing the *z* threshold from resting-state and task-based language maps. In the threshold ranges where Dice coefficients reached maximum values (*z* = 2 for rs-fMRI; *z* = 3.25 for task-fMRI and *z* = 2.5 for taskIC-fMRI, see **Figure 5**, upper panel), we observed small but non-significant increases in the Dice coefficient from 0.248 to 0.256 in the GLM analysis [*t*(14) < 1] and from 0.298 to 0.305 in taskIC-fMRI [*t*(14) = 1.27, *p* = 0.22]. Though not significant, we note a tendency toward higher thresholds in task-fMRI, as the mean Dice coefficient is increased off-diagonally. Importantly, at these thresholds, the masks of the three methods did not differ in size [rmANOVA, *F*(2,28) = 2.93, *p* = 0.09; rs-fMRI 16418 ± 980 voxels, task-fMRI 23574 ± 3368 voxels, and taskIC-fMRI 20280 ± 1565 voxels].

We further compared the specificity and sensitivity for rs-fMRI, task-fMRI and taskIC-fMRI at higher *z* thresholds (**Figure 5**, lower panel). Resting-state masks had higher specificity for all thresholds when compared to task-related methods (main effects of size, *p*s < 0.001; rs-fMRI vs. task-fMRI, *p*s < 0.001, rs-fMRI vs. taskIC-fMRI, *p*s < 0.001). No differences were observed between task-fMRI and taskIC-fMRI (*p*s > 0.23). For sensitivity, there were no statistical differences between the three methods in thresholds of *z* = 2 and *z* = 3 (*p*s > 0.18). At thresholds *z* = 4 and *z* = 5, there were main effects of method [for *z* = 4: *F*(1.6,22.5) = 3.69, *p* = 0.049, $\eta_{p}^{2}$ = 0.21; for *z* = 5: *F*(1.5,21.4) = 3.95, *p* = 0.044, $\eta_{p}^{2}$ = 0.22]. Task-fMRI had higher sensitivity values than taskIC-fMRI (all *p*s < 0.05). Most importantly, at these thresholds, the sensitivity of rs-fMRI did not differ from task-based methods (*p*s > 0.11). At the threshold *z* = 6, a main effect of method was also observed [*F*(1.4,20.3) = 5.11, *p* = 0.024, $\eta_{p}^{2}$ = 0.27]. Rs-fMRI (1781) and task-fMRI (1651) had higher sensitivity values than taskIC-fMRI (996, *p* = 0.016 and *p* = 0.048, respectively).

### Influence of Dimensionality on the Language Networks

**FIGURE 6:**
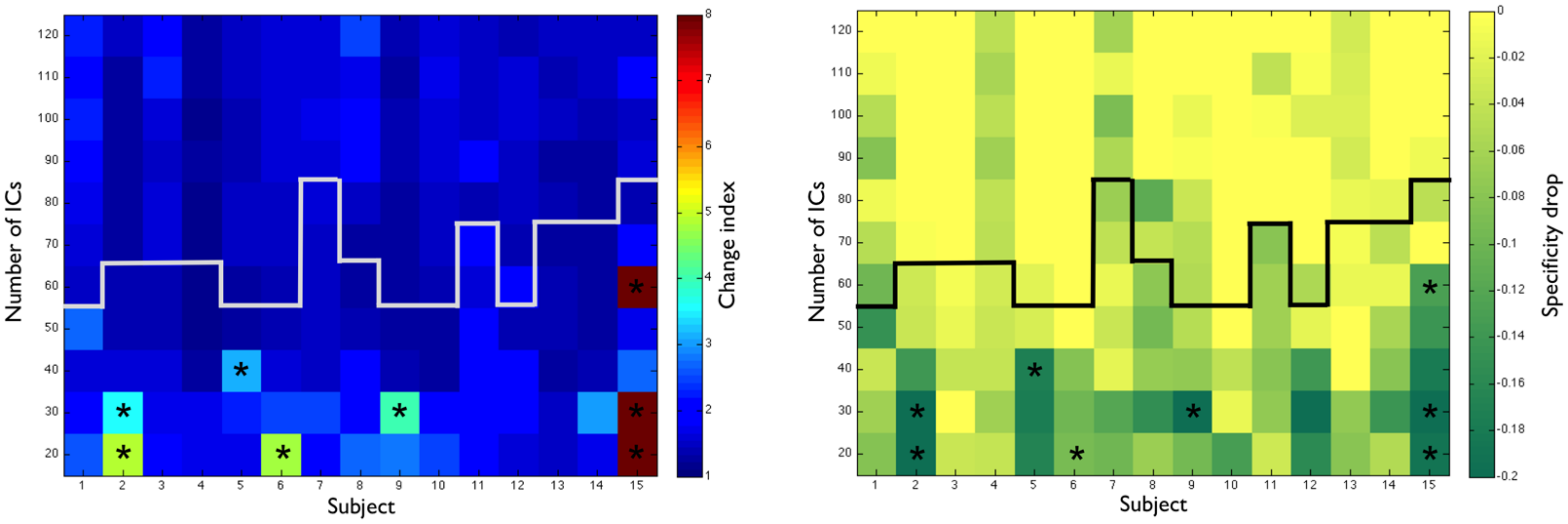
**(Left panel)** Change index [1/(spatial correlation x temporal correlation)] between the original resting-state IC and ICA dimensionalities from 20 to 120. The white line represents the original dimensionality as determined by MELODIC. **(Right panel)** Drop in specificity values (specificity at the original dimensionality minus specificity at each new dimensionality). The black line represents the original dimensionality as determined by MELODIC. ^∗^ Asterisks signal ICs with a change index larger than 3.

To examine the impact of ICA dimensionality in the extraction of the language networks, we ran ICAs with a fixed number of ICs from 20 to 120 ICs (**Figure 6**, left panel). At higher dimensionalities (>50), the language IC was stable with only minor changes. This indicates that the extraction of the language IC was robust within this range (note that this range includes values from the automated dimensionality estimation). At lower dimensionalities, however, noticeable changes — change index values above 3 — were observed in 5 of 15 subjects. Maps with these ICs can be inspected in Supplementary Figure S2. These changes were associated with a decrease in specificity as compared to the specificities in the original dimensionality (**Figure 6**, right panel), indicating that language ICs were merging with ICs that were unrelated to language. For one subject (Case 2), the language IC merged with a right-hemisphere component that resembled a right-lateralized language network; however, as neither task-fMRI nor taskIC-fMRI results showed this specific right-hemisphere activity, this result was probably artefactual.

## Discussion

The goal of this study was to examine whether resting-state networks can identify language regions as do task-based methods, in subjects with brain pathology. We compared resting-state language networks with the language maps obtained by task execution in tumor and epilepsy patients indicated for pre-surgical planning. Three main findings were revealed. First, we showed that ICA together with a template-matching procedure is well suited to extract and identify the language resting-state network. Second, a task-free paradigm was able to provide similar results to those identified by task-execution, particularly in critical language areas. Third, and importantly, resting-state networks were as sensitive as task-based maps, and had the advantage of higher specificity. This study forms the first demonstration that resting-state protocols are effective to map language regions in patients with brain lesions.

### Automated Classification of Resting-State Independent Components for Language

One of the challenges of using data-driven methods such as ICA is the classification of language components in subjects with brain lesions. Due to changes in brain anatomy, template-matching algorithms face methodological challenges such as coping with the dislocation of critical brain regions and the presence of artifacts at the periphery of the lesions (Duffau, 2005). Template-matching procedures using group-derived templates are able to filter the ICA extracted components into a shorter repertoire (Tie et al., 2014). This enables experts to correctly identify the language networks from a reduced number of options. However, this implies having group data available, and the results obtained from such an approach may vary depending on the sample characteristics. Most importantly, and to the best of our knowledge, no study attempted to perform the classification of language ICs in subjects with brain lesions. In this study, we performed a template-matching procedure using a comprehensive set of language ROIs that correspond to frequently activated brain regions during task-execution (Fedorenko et al., 2010). These ROIs were selected due to their large extent, in order to cope with the dislocation of brain regions due to lesions, while providing a good estimate of language localization in the brain.

Our classification results suggest that a template-matching procedure performs well in subjects with brain lesions. Using this approach, the components of 12 out of 15 subjects were correctly classified, and the remaining three subjects had their language components ranked as the second best fit option. Although the method did not yield perfect accuracy, it allowed us to reduce the number of components to a handful of alternatives, thus significantly lessening the workload and expertise needed to implement this procedure in clinical practice. It is important to note that we used a left-sided language template, and this yielded good results with our patients. However, in patients with atypical language lateralization and in the absence of task-fMRI to compute laterality indices, left- and right-sided language templates should be used to ensure correct identification of the language network.

An important limitation of ICA is the risk of splitting the language network into two or more ICs. In our study, it was always possible to select one representative language component. We confirmed this result by examining the stability of the language ICs in a wide range of dimensionalities (20–120) and found no evidence of split language ICs. Our result is at odds with a previous study using ICA with a fixed number of 40 components that found for each subject a statistical mode of two components for each subject (Tie et al., 2014). Our method had one important difference: we used a Laplacian estimation of the ideal number of ICs instead of choosing a fixed number for all subjects. This was done to avoid overfitting or underfitting the data, thus establishing the number of ICs based on the intrinsic properties of the data (Minka, 2000; Beckmann and Smith, 2004). Our findings suggest that optimally choosing the number of components may partially solve the problem of separating language networks into two or more components. We were able to correctly narrow down the language components for all subjects with good accuracy, when compared to expert manual classification. However, less than 100% accuracy and the risk of splitting the language network make it imperative that the pre-selected ICA components are carefully visually inspected.

### Comparison Between Resting-State and Task-Based Language Components

Our results showed that rs-fMRI maps had a fair overlap with the maps obtained from traditional task-fMRI using GLM methods. We observed a Dice coefficient of 0.248 for whole-brain analysis when using a threshold of *z* = 2.3, and analysis restricted to language regions of interest increased the Dice coefficient to 0.458, suggesting that the overlap between task and resting-state methods was significantly higher within critical brain regions that are typically involved in language processing. This is convergent with previous results from Tie et al. (2014), who found a similar overlay in a sample of healthy subjects. We extend these findings by showing that it is also reproducible in subjects with brain pathology, therefore providing additional evidence that this technique may be feasible in clinical settings. These results also closely mirror those observed in studies comparing both techniques on the somatosensory cortex in subjects with brain lesions, where a moderate overlap between resting-state and task-evoked fMRI was observed (Kokkonen et al., 2009; Zhang et al., 2009; Rosazza et al., 2014). Our results indicate that language, similarly to the somatosensory areas, can be localized using resting-state methods.

When using ICA to extract task-related activity, the overlay between task and resting-state fMRI was improved. This was expected as ICA methods can separate artifacts and non-language related activity from the obtained language maps (Tie et al., 2008; Robinson and Schopf, 2013), thus excluding potential confounds from the data. Likewise, in this condition we used the same threshold procedure as for resting-state analysis (mixture-modeling at equal weights for false-positives and false-negatives), thus selecting the threshold as a result of the model fit instead of assuming a fixed value. This strategy is more reliable for the selection of thresholds in single-subject analyses (e.g., Gorgolewski et al., 2012), reducing the potential bias induced by a fixed *z* threshold. Notably, the Dice coefficient was increased when using ICA to extract task-activity in the whole-brain analysis but not when restricted to language ROIs. This suggests that the presence of activity outside critical brain regions identified by the GLM methods influenced the overlay between task and resting-state results. With ICA-based methods we observed less activity outside of the critical brain regions, which partially explains this result. Taken together, our results indicate that the overlay results are influenced by the methods used, and that a more conservative approach improves the similarity between resting-state and task-based techniques.

### Specificity and Sensitivity for Resting-State and Task-Based Methods

Comparing resting-state networks to task-based masks assumes that task masks should be sensitive and specific enough for language mapping. Due to performance deficits, it is possible that the task mapping results are poor, thus influencing the Dice coefficient. One way to address this problem is to compare the results of the resting-state maps and both task-maps to a third reference, and measure how well they correspond. We did so by comparing rs-fMRI, task-fMRI, and taskIC-fMRI masks to Fedorenko et al.’s (2010) validated language ROIs. The extracted resting-state language networks showed larger specificity than both task-based methods. As previously discussed, because task-based activation is typically not restricted to the critical language regions (i.e., it contains regions involved in visual processes, attention and working-memory), this is expected. Thus, resting-state analysis is able to extract language regions more selectively than task-based methods, allowing to identify the critical brain regions for language while excluding non-language-related processes. In a neurosurgical setting, however, sensitivity is the most important attribute as the risk of false-negatives may lead to patient injury. In this study, we did not observe differences in sensitivity between task and resting-state based methods. We did observe a trend toward larger sensitivity in task-based GLM methods. However, given that the task-based maps were significantly larger in size than resting-state maps and because the sensitivity is likely to be linearly correlated with the size of the mask (e.g., Kristo et al., 2014), true advantages in the sensitivity of task-based methods are unlikely. This is observable in **Figure 5**, lower panel, where differences between the sensitivity of task-fMRI and rs-fMRI disappear at higher thresholds. The overlay maps shown in **Figure 4** further corroborate this, because they reveal a larger spatial extent of areas identified through task-based protocols than of resting-state language networks, yet at a cost in specificity.

In a previous comparison between task and resting-state language maps in healthy subjects, higher values of sensitivity for resting-state networks were observed, but there were no differences in specificity (Tie et al., 2014). This is in contrast to our findings. The contradictory results are likely related to differences in the methods used. Because our ROIs were large enough to encompass putative anatomical distortions in brain lesion patients, high values of sensitivity were expected. Thus, it is possible the language ROIs employed here could not successfully differentiate between the true sensitivity of the two methods. In addition, because our sample consisted of patients with brain lesions, we expected more artefactual activations in the data. This led to noisy task-based results, particularly when using GLM, which could in turn reflect in lower specificity values. In the future, it will be important to compare task and resting-state methods to DCS to further clarify the differences in sensitivity observed in the present study and in previous ones.

### Clinical Feasibility of Resting-State Protocols for Language Mapping

Our results provide evidence of a partial overlay between resting-state and task-fMRI. However, the relatively low Dice coefficients of 0.248 for task-fMRI and 0.298 for taskIC-fMRI in the whole-brain analysis do not warrant to consider resting-state as a stand-alone alternative to task-fMRI for clinical purposes. Key aspects when comparing Dice coefficients between task and resting-state protocols in clinical populations are the task used and subjects’ performance. Assuming task-related activity as ground-truth is sub-optimal because the brain regions identified will depend on the task itself and the ability of the subject to perform it. Consequently, a perfect match between both techniques is not to be expected. We used a verb-to-noun generation paradigm, which is common in neurosurgical language mapping due to ease of application and robustness. However, it is clear that this task does not capture language in its entirety. Also, resting-state networks consist of brain regions that share similar temporal patterns during rest. The correspondence between the extracted networks and their functional roles remains largely undetermined, except for the spatial resemblance with the task-derived homologues. This raises important questions as to whether brain regions that are functionally interconnected are indeed active during task-execution. To assess directly the functional role of these brain regions, more invasive procedures such as DCS have to be used. To our current knowledge, very few studies compared resting-state maps with this technique. Mitchell et al. (2013) did so with a series of epilepsy and brain tumor patients. They observed good correspondence between positive DCS sites and motor and language functional maps. However, no task-data was available, and so to which extent both techniques converge is still unknown. Future studies comparing both methods with DCS will have to address this question.

One of the advantages of the methods employed here is that they are data-driven. Because language is a complex system with several brain regions, the need to select seed regions to perform the alternative seed-based correlation analysis (SBCA) procedure (e.g., Zhu et al., 2014) is a considerable limitation. This becomes even more evident in subjects with clinical pathologies where the selection of a good seed region may be challenging because of brain distortions and brain plasticity. ICA has the advantage of not requiring *a priori* definition of functional regions, and this makes it an attractive tool to study functional connectivity. On the other hand, due to the absence of a task in a largely unconstrained environment, it is critical to assess whether ICA language maps can be reproduced in the same subjects in different time-periods and across centers. Recent findings by Kristo et al. (2014) indicate that task-based approaches focusing on the somatosensory region may be better suited for mapping motor functions, because they are more reproducible across sessions. When controlling for threshold and size differences, and by performing SBCA, Kristo et al. (2014) observed higher test-retest indices in task-based mappings of the motor cortex than in resting-state mappings. However, an earlier study by Mannfolk et al. (2011) had showed similar test–retest indices between task and resting-state somatosensory cortex maps when using ICA. Taken together, both findings suggest that mapping results using data-driven approaches are less variable for single-subject mapping when compared to SBCA. As for language mapping, Zhu et al. (2014) found good short term (45 min apart) and long term (5–16 months apart) reproducibility of the language maps in healthy subjects using SBCA. No study examined test–retest validity of rs-fMRI in language using ICA. This will be crucial in determining whether resting-state approaches may be fit for clinical practice. Importantly, previous studies used healthy subjects. With patients, and in complex cognitive functions such as language, it is likely that task-performance deficits would outweigh the reduced reproducibility identified by Kristo et al. (2014). Interestingly, Zhang et al. (2009) reported a case study where resting-state methods were able to map motor regions and a task-based approach was not. In our study we observed that task-based language maps showed low specificity and poor inter-subject coherence, which suggests suboptimal language mapping. In contrast, resting-state maps showed better specificity, comparable sensitivity and overall better inter-subject coherence, particularly in the identified language regions. Thus, although resting-state protocols present some limitations, they also have advantages that may be critically relevant in clinical settings.

The present study highlights some of the potential advantages of resting-state functional connectivity analyses over traditional task-based approaches. A task-free procedure offers several advantages for clinical practice: as the subject does not have to perform a task, it may be more widely used across a wide spectrum of patients, including those with task-performance deficits, children, subjects with hearing or vision problems, and potentially subjects under anesthesia. The duration of the MRI acquisition protocol is short (here 7 min 10 s), thus easily adaptable into each patient’s scanning schedule. Because of its time-efficiency it can also be used as a confirmatory measure for task-based fMRI, particularly in challenging subjects. Finally, unlike task-based fMRI, which needs to be planned previously and serves only one purpose, resting-state data allows *post hoc* analyses of several brain functions. Hence, resting-state data may be used for other purposes, such as to identify other well-known networks (e.g., somatosensory network, default mode network, etc.). It also provides a tool to study functional connectivity, and thus can be used for other neurosurgical applications such as identifying epileptic focus (Bettus et al., 2010; Stuﬄebeam et al., 2011), targeting deep brain stimulation sites (Smith et al., 2012), or predicting neuropsychological outcome after surgery (Negishi et al., 2011; McCormick et al., 2013).

### Limitations and Prospects

This study presents some limitations. Our sample is small and heterogeneous and we could not measure the impact of the lesion type and localization in the feasibility of using rs-fMRI for language mapping. We also lack DCS data to support our findings. Thus, even though we observed a fair overlay between resting-state and task-derived maps, our results do not indicate whether the regions that are not in agreement between the two techniques play a relevant role in language processing and consequently represent a risk of future deficits in case of brain tissue removal. Regarding the order of the protocols, resting-state was performed after the task-based fMRI in order not to interfere with the clinical examination. However, most of the subjects (10 out of 15) had other routine exams (e.g., DTI, motor mapping) performed in between the task and rs-fMRI protocols. Although unlikely, we cannot discard the possibility that resting-state data might have been affected by the task-based protocol. Another limitation is that the classification of language ICs was inspected by one expert only. We note though that a previous study (Tie et al., 2014) with five independent experts showed good inter-rater agreement in the classification of the language component in 17 of 18 subjects. Our sensitivity and specificity measures might have been influenced by the selection of ROIs. Despite previous validation of the masks in healthy subjects (Fedorenko et al., 2010), assuming ROIs as ground-truth does not permit to study the true sensitivity and specificity of the language mapping, but rather how well the results fit with previous evidence. Finally, we also lack a behavioral correlate of task-performance to verify that the subjects performed the language task correctly. In the future it will be interesting to see if subject performance can explain the poor overlay between techniques, that is, if the task-based maps are driving the lack of a better fit between resting-state and task maps.

## Conclusion

Our results suggest that resting-state methods are a promising tool for preoperative mapping of language. By combining data-driven techniques without *a priori* assumptions, we were able to successfully map language brain regions in patients. We found that by using a template-matching procedure it was possible to reduce the number of components to a few alternatives. The adopted procedure has the advantage of being easy and fast to perform. It is readily available by using free software and previously published template maps. The absence of a task reduces possible confounds (e.g., visual activation), encourages *post hoc* analysis of other brain networks, and improves the quality of the language mapping in subjects with poor task-performance. In conclusion, and in line with the proposals of other authors (Zhang et al., 2009; Mitchell et al., 2013; Tie et al., 2014), this study provides evidence that resting-state networks may play a role in the future of preoperative planning. Although further research is needed, the use of resting-state protocols is likely to grow in the future, especially with increasing scanner field strengths and improvements in data-processing methods.

## Author Contributions

PB, DS, SC, and SS designed the study. SK collected the data. DS, RP, and SS supervised data-analysis and SD contributed to data-analysis. PB and SC wrote the manuscript. All authors approved the final manuscript.

## Conflict of Interest Statement

The authors declare that the research was conducted in the absence of any commercial or financial relationships that could be construed as a potential conflict of interest.

## Footnotes

1 The equation is as follows: *Dice* = $\frac{2\left|X\cap Y\right|}{X+Y}$

2 These subjects were cases 4, 9, and 11 (2 tumors, 1 epilepsy, see **Table 1**).
