# Supplementary material for: Resting-State Functional Magnetic Resonance Imaging for Language Preoperative Planning
Source: Front Hum Neurosci. 2016 Feb 1;10:11. doi: 10.3389/fnhum.2016.00011 (PMC4740781; doi:10.3389/fnhum.2016.00011)
Supplement: Supplementary file 1 [file Data_Sheet_1.PDF]

Supplementary Material

Resting-state functional magnetic resonance imaging for language preoperative planning

Paulo Branco, Daniela Seixas, Sabine Deprez, Silvia Kovacs, Ronald Peeters, São Luís Castro*, Stefan Sunaert

Correspondence: São Luís Castro: slcastro@fpce.up.pt

Supplementary Data

Supplementary Figures

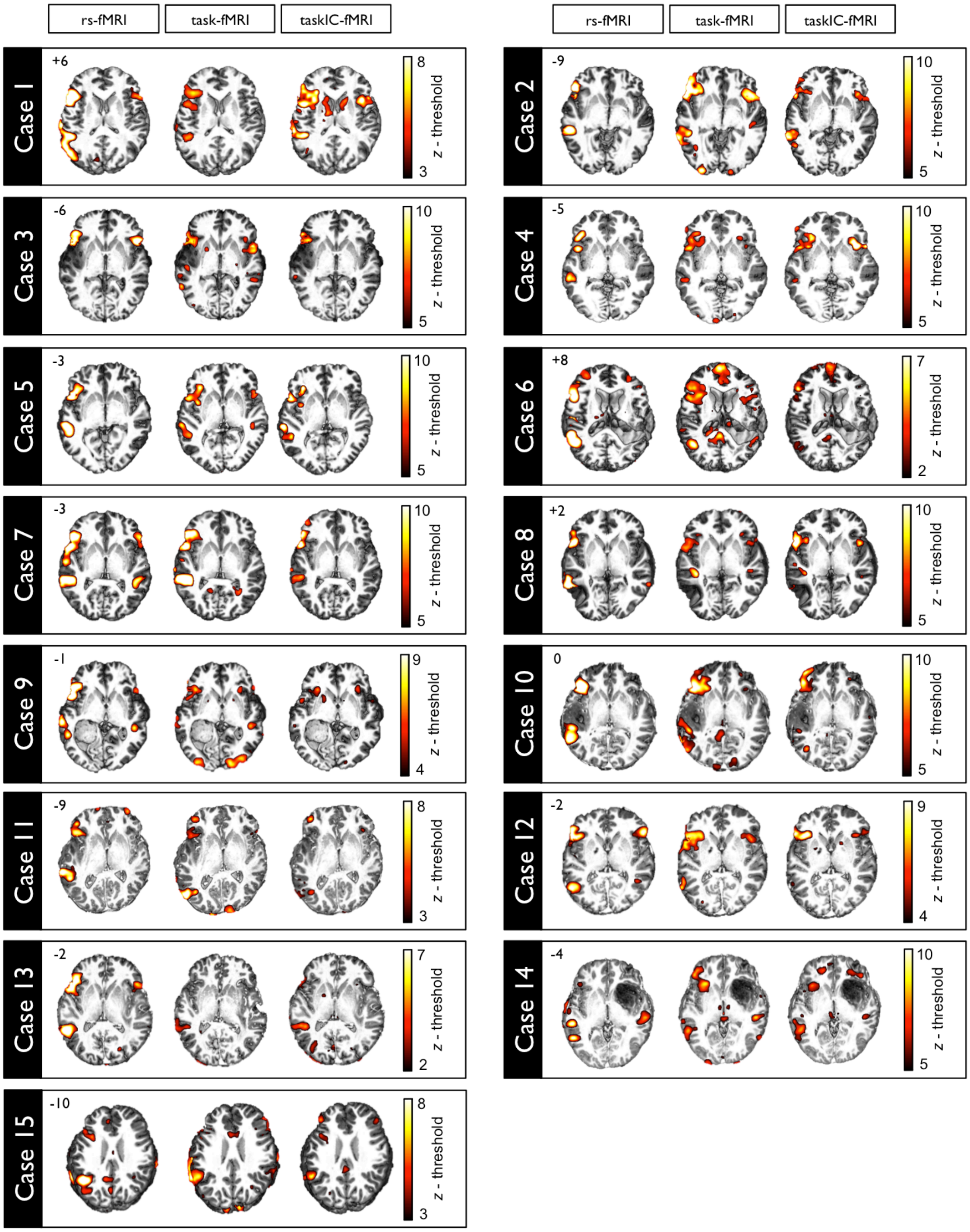

Supplementary Figure S1. Representative axial slices of rs-fMRI, task-fMRI and task IC-fMRI language mapping for all subjects. Images are shown in neurological convention (left is left). MNI coordinates are shown at the upper-left side of the image. For illustration purposes, different thresholds were used for each subject, although they were kept constant between conditions. In Case 14, taskIC-fMRI is displayed with a threshold of $z > 3$ unlike rs-fMRI and task-fMRI ($z > 5$).

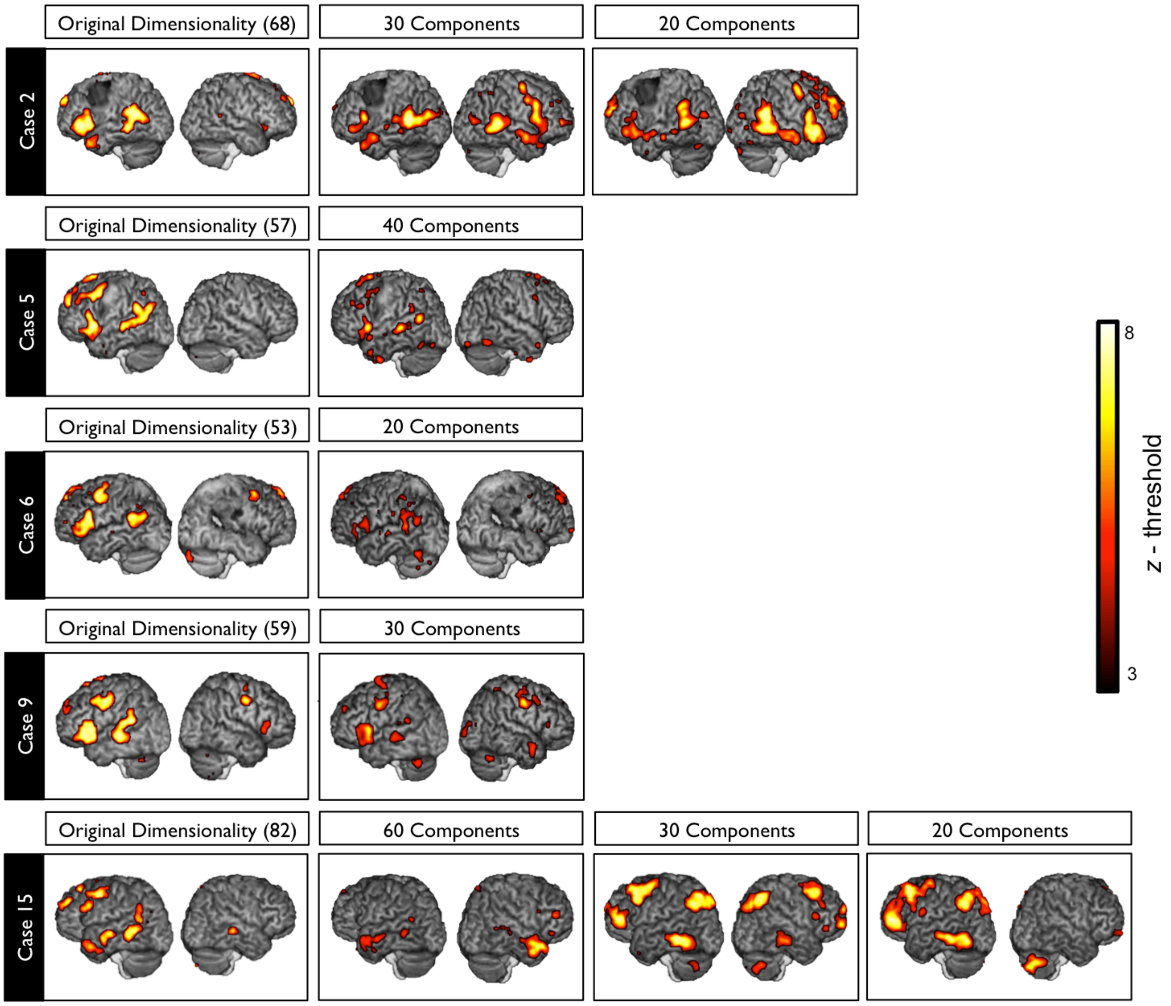

Supplementary Figure S2. Changes from resting-state language networks at different ICA dimensionalities (change index > 3), with the language network at the original dimensionality for comparison. Threshold was set at z > 3 for illustration purposes.
